# Competing risks analyses for recurrence from primary breast cancer

**DOI:** 10.1038/sj.bjc.6690240

**Published:** 1999-03

**Authors:** J W Chapman, E B Fish, M A Link

**Affiliations:** Henrietta Banting Breast Centre, Women's College Hospital, University of Toronto, 60 Grosvenor Street,5th floor, Toronto, M5S 1B6, Ontario, Canada

**Keywords:** breast cancer, competing risks, prognostic factors

## Abstract

The effects of prognostic factors on local, regional or distant metastasis are standardly assessed separately. Competing risks analyses may be used to assess simultaneously the effects of factors on different types of first recurrence. Data for a cohort of 678 primary invasive breast cancer patients accrued between 1971 and 1990, updated to 1995, included type of first recurrence (local, regional, distant). We investigated the effects of the traditional factors of age, tumour size, nodal status, ER, PgR, adjuvant therapy (hormones, chemotherapy, radiotherapy) on type of recurrence and time to recurrence for all patients and for those aged ≥ 65. For all ages of patients, there were five factors with significant associations with type or time to first recurrence. Adjuvant radiation was the only factor which had an effect (*P* ≤ 0.05) on the type of first recurrence: being associated with a reduction in local recurrence. Age, nodal status, tumour size and adjuvant chemotherapy all had significant associations across all types of first recurrence, and in particular with time to recurrence for both local and distant metastasis. This indicates a potential lack of independence in these end-points. For patients ≥ 65 years of age, there were no factors which differentially affected type of recurrence, while only nodal status and tumour size had significant associations with time to recurrence. Analyses were used to assess simultaneously the effects of traditional prognostic factors and treatment options on type of first recurrence and time to first recurrence. The extension to evaluations with newer prognostic factors would expedite the determination and mode of biologic activity for such factors. © 1999 Cancer Research Campaign


*British Journal of Cancer* (1999) **79**(9/10), 1508–1513
© 1999 Cancer Research Campaign
Article no. bjoc.1998.0240

### Competing risks analyses for recurrence from primary breast cancer

**JW Chapman, EB Fish and MA Link**

Henrietta Banting Breast Centre, Women's College Hospital, University of Toronto, 60 Grosvenor Street, 5th floor, Toronto, Ontario, Canada M5S 1B6

**Summary** The effects of prognostic factors on local, regional or distant metastasis are standardly assessed separately. Competing risks analyses may be used to assess simultaneously the effects of factors on different types of first recurrence. Data for a cohort of 678 primary invasive breast cancer patients accrued between 1971 and 1990, updated to 1995, included type of first recurrence (local, regional, distant). We investigated the effects of the traditional factors of age, tumour size, nodal status, ER, PgR, adjuvant therapy (hormones, chemotherapy, radiotherapy) on type of recurrence and time to recurrence for all patients and for those aged ≥ 65. For all ages of patients, there were five factors with significant associations with type or time to first recurrence. Adjuvant radiation was the only factor which had an effect ($P \leq 0.05$) on the type of first recurrence: being associated with a reduction in local recurrence. Age, nodal status, tumour size and adjuvant chemotherapy all had significant associations across all types of first recurrence, and in particular with time to recurrence for both local and distant metastasis. This indicates a potential lack of independence in these end-points. For patients ≥ 65 years of age, there were no factors which differentially affected type of recurrence, while only nodal status and tumour size had significant associations with time to recurrence. Analyses were used to assess simultaneously the effects of traditional prognostic factors and treatment options on type of first recurrence and time to first recurrence. The extension to evaluations with newer prognostic factors would expedite the determination and mode of biologic activity for such factors.

**Keywords:** breast cancer; competing risks; prognostic factors

Medical decisions about type of primary surgery and adjuvant therapy in breast cancer are influenced by a patient's expected prognosis. Reduction in death from breast cancer is the ultimate goal in both preventive and therapeutic strategies. We have used competing risk analyses (Fish et al, 1998) to investigate the effects of surgical procedure, adjuvant therapy and traditional prognostic factors for breast cancer (age, tumour size, nodal status, ER and PgR) on whether a patient will die from breast cancer or some other cause, as well as the time to death.

The effects of factors on recurrence at local, regional or distant sites are usually assessed separately. In part, one might view local recurrence as being controllable with the modalities of surgery and radiotherapy. Regional and distant metastases are more commonly viewed as potential risk for breast cancer death.

In the context of recurrence, Gelman et al (1990) discussed reasons why, even in a randomized clinical trial, the assessment of treatment effects may not be straightforward for recurrence end-point(s). The standard statistical assumption of independence of end-points such as local and distant recurrence may be violated to such an extent that a competing risks analysis is better used under such circumstances. For instance, analytic assumptions about unobtainable recurrence information could lead to it being impossible to ascertain precisely whether adjuvant radiation was a beneficial adjunct for the local control of breast cancer.

Veronesi et al (1995) demonstrated with competing risks analyses of a large cohort of 2233 patients, all of whom had received the breast conserving procedure of quadrantectomy, that there was a significant lack of independence between the development of local and distant recurrence; patients who developed a local recurrence had a hazard of relapsing from distant metastases that is 4.62 times greater than that for patients who did not develop a local recurrence. Further, they showed that factors could differentially affect the development of local and distant recurrence.

The analysis background for a competing risks assessment may be found elsewhere: Allison (1995) is an introductory text while Kalbfleisch and Prentice (1980) and Cox and Oakes (1984) contain more advanced statistical presentations. The particular hierarchical approach employed in this paper is illustrated for lung cancer data in Lagakos (1978).

We report here the results of competing risks analyses to assess simultaneously the effects of traditional factors on recurrence at local, regional and distant sites for a cohort of primary breast cancer patients, many of whom received minimal surgery. Additionally, most patients did not receive adjuvant therapy; this was particularly true for the elderly.

### MATERIALS AND METHODS

### Patients

All the patients assessed and followed were from the surgical practice of EBF. They presented with unilateral primary invasive breast cancer, stage 1–3, with no previous history of carcinoma, except possibly in situ cervix or non-melanoma skin. The cohort of 678 consecutive patients meeting these criteria were accrued between 1971 and 1990. The numbers of patients operated on by calendar year, and type of surgery have been reported elsewhere (Fish et al, 1998). The follow-up to 1995 was complete for 91% of

*Received 3 June 1998*
*Revised 25 September 1998*
*Accepted 9 October 1998*

*Correspondence to:* JW Chapman

**Table 1** First recurrence by age group

| Patient group | No. of cases | Type of first recurrence: Local | Regional | Distant | First recurrence | *P*-value[a] |
|---|---|---|---|---|---|---|
| < 65 | 471 | 77 (16%) | 16 (3%) | 81 (17%) | 174 (37%) | 0.28 |
| ≥ 65 | 207 | 25 (12%) | 5 (2%) | 27 (13%) | 57 (28%) | |

[a]Based on Wilcoxon (Peto–Prentice) test statistic.

patients. The median follow-up for those alive was 8.2 years for all patients, and 7.7 years for those over 65 years of age.

### Surgical procedure

In all, 366 patients received a lumpectomy; 119 were clinically node negative (Nx) and elected to have no axillary dissection, while 247 had an axillary dissection and were designated to be N– or N+. Lumpectomy is defined as a surgical attempt to remove the entire tumour and enough surrounding tissue to ensure the excision was adequate. Usually, about 2 cm of normal breast tissue was removed in each direction and, nearly always, the underlying pectoralis fascia. If the tumour was near the skin, a thin ellipse of skin was taken to indicate the anterior margin. The specimens were inked and they were examined by a pathologist in gross at the time of surgery, and on paraffin sections to assess the borders.

A mastectomy was performed on 312 patients (simple or subcutaneous, 50 patients; modified radical, 262 patients). The regional recurrence rates for the mastectomy patients were 0% (0/112) for N–, 4% (2/50) for Nx, and 1% (2/150) for N+ ($P = 0.11$); the corresponding data for those ≥ 65 years of age is 0% (0/32) for N–, 4% (1/26) for Nx, and 3% (1/30) for N+ ($P = 0.52$).

### Effects of covariates

The event of interest was the first recurrence which was ascertained to be local, regional or distant. A patient's time on study was the time until first recurrence (event), time until death from another

**Table 2** First recurrence by factor subgroup

| Factor | No. of cases | Type of first recurrence: Local | Regional | Distant | First recurrence | *P*-value[a] |
|---|---|---|---|---|---|---|
| Age | | | | | | |
| ≤ 49 | 197 | 40 (20%) | 3 (2%) | 29 (15%) | 72 (37%) | 0.51 |
| 50–64 | 274 | 37 (14%) | 13 (5%) | 52 (19%) | 102 (37%) | |
| ≥ 65 | 207 | 25 (12%) | 5 (2%) | 27 (13%) | 57 (28%) | |
| Tumour size (cm) | | | | | | |
| ≤ 2 | 363 | 46 (13%) | 11 (3%) | 46 (13%) | 103 (28%) | < 0.001 |
| [2–5] | 266 | 48 (18%) | 8 (3%) | 48 (18%) | 104 (39%) | |
| > 5 | 32 | 5 (16%) | 1 (3%) | 10 (31%) | 16 (50%) | |
| Nodal status | | | | | | |
| N– | 251 | 24 (10%) | 0 (0%) | 32 (13%) | 56 (22%) | < 0.001 |
| Nx | 169 | 33 (20%) | 13 (8%) | 15 (9%) | 61 (36%) | |
| N+ | 258 | 45 (17%) | 8 (3%) | 61 (24%) | 114 (44%) | |
| ER | | | | | | |
| < 10 fmol/mg protein | 115 | 22 (19%) | 4 (3%) | 15 (13%) | 41 (36%) | 0.21 |
| ≥ 10 fmol/mg protein | 472 | 68 (14%) | 14 (3%) | 83 (18%) | 165 (35%) | |
| PgR | | | | | | |
| < 10 fmol/mg protein | 90 | 16 (18%) | 1 (1%) | 13 (14%) | 30 (33%) | 0.38 |
| ≥ 10 fmol/mg protein | 353 | 54 (15%) | 9 (3%) | 57 (16%) | 120 (34%) | |
| Adjuvant radiation | | | | | | |
| No | 517 | 91 (18%) | 18 (3%) | 78 (15%) | 187 (36%) | 0.12 |
| Yes | 159 | 11 (7%) | 3 (2%) | 30 (19%) | 44 (28%) | |
| Adjuvant chemotherapy | | | | | | |
| No | 572 | 87 (15%) | 19 (3%) | 86 (15%) | 192 (34%) | 0.57 |
| Yes | 104 | 15 (14%) | 2 (2%) | 22 (21%) | 39 (38%) | |
| Adjuvant hormones | | | | | | |
| No | 556 | 91 (16%) | 19 (3%) | 81 (15%) | 191 (34%) | 0.73 |
| Yes | 118 | 11 (9%) | 2 (2%) | 27 (23%) | 40 (34%) | |

[a]Based on Wilcoxon (Peto–Prentice) test statistic (Prentice and Marek, 1979).

© Cancer Research Campaign 1999

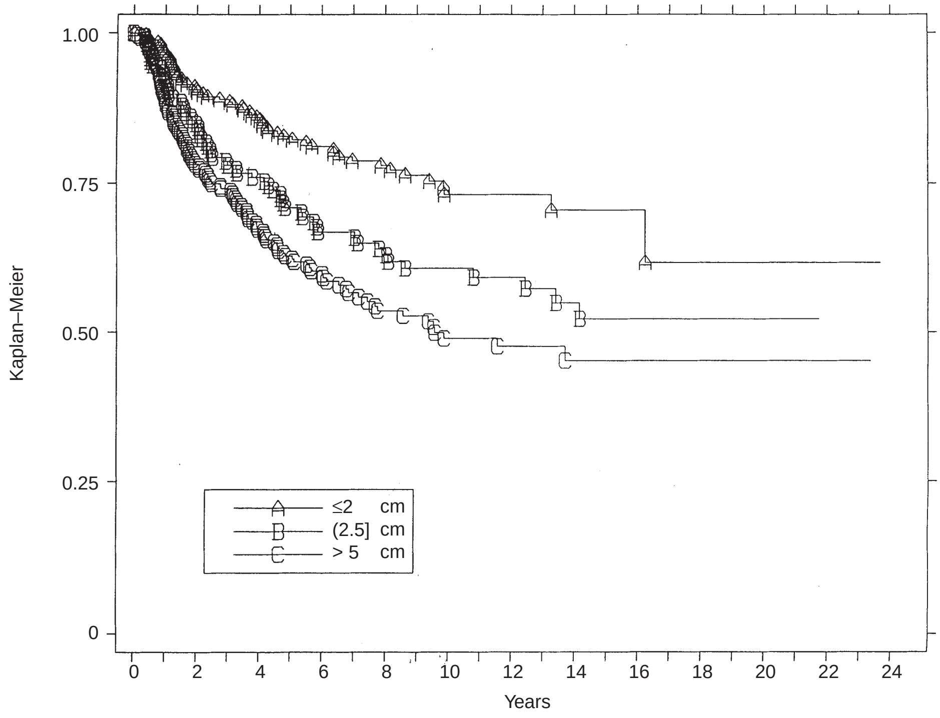

**Figure 1** Disease-free survival by tumour size

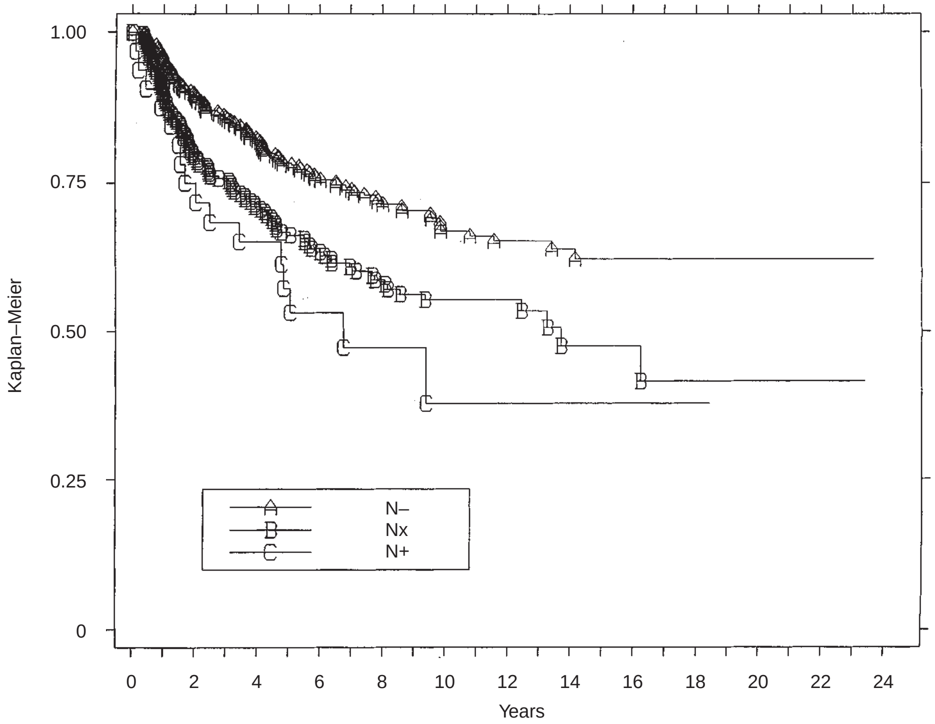

**Figure 2** Disease-free survival by nodal status cause (patient was 'censored' at time of death), or length of follow-up (patient was alive and well – 'censored'). The date for first recurrence always preceded a patient's death from breast cancer.

We investigated the multivariate effects of the covariates on recurrence in the following units: age (in years), tumour size (cm), nodal status (N–, Nx, N+), oestrogen and progesterone receptors (ER and PgR; fmol/mg protein), adjuvant radiotherapy (no, yes), adjuvant hormonal therapy (no, yes), and adjuvant chemotherapy (no, yes) on time to first recurrence. With the exception of nodal status where Nx patients are known to be clinically node negative,

 *© Cancer Research Campaign 1999*

**Table 3** Cox stepwise regression for first recurrence of any type

| Factors in best models | $-\hat{\beta}$/s.e.[a] | *P*-value |
|---|---|---|
| All patients | | |
| Tumour size | – 4.28 | < 0.001 |
| Nodal status | – 4.36 | < 0.001 |
| Adjuvant chemotherapy | 3.16 | 0.002 |
| Age | 3.00 | 0.003 |
| Adjuvant radiotherapy | 2.56 | 0.01 |
| PgR | 1.75 | 0.08[b] |
| Patients ≤ 65 years of age | | |
| Nodal status | – 2.97 | 0.003 |
| Tumour size | – 2.73 | 0.01 |

[a]A positive/negative coefficient suggests a positive/negative effect for larger values of the factor on a type of recurrence. [b] – 2 log *R* *P*-value for addition of factor to model = 0.05.

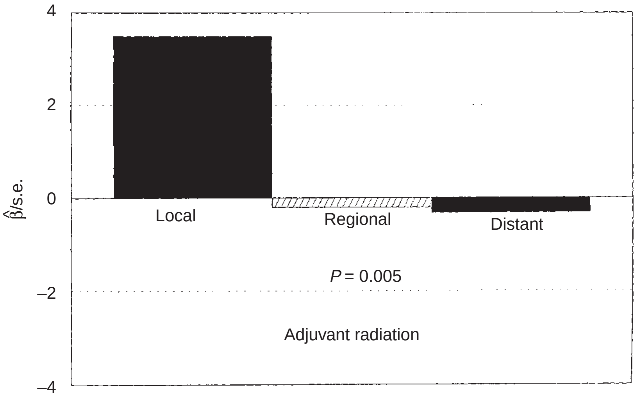

**Figure 3** Covariates with significant effects on type of first recurrence – all ages. The standardized coefficients, $\hat{\beta}$/s.e., permit a comparison of the potential effects across factors. A positive/negative coefficient suggests a positive/negative effect for larger values of the factor on a type of recurrence patients with missing data for any factor were not included in the multivariate analyses. Owing to the large amount of missing data for PgR, analyses were performed both with and without PgR; the only results reported here are those with PgR, since it was included in the best models. The use of the larger data set, obtained by excluding PgR, did not substantially alter the competing risks results for other factors.

Univariate Kaplan–Meier plots were made, and the Wilcoxon (Peto–Prentice) test statistic (Prentice and Marek, 1979) was used to assess the univariate effects for all ages of patients, and for those patients ≥ 65 years of age. A split was made at age 65 due to differences in treatment recommendations based on likely aggressiveness of breast cancer, ability to mammographically detect recurrence, and overall life expectancy.

We investigated the multivariate effects of the covariates with Cox and log-normal (Kalbfleisch and Prentice, 1980) forward stepwise regressions, performed for the whole patient group and those ≥ 65 years of age. The model improvement for the addition of *k* factors to the model was assessed with the likelihood ratio criterion; where *R* is the likelihood ratio, $-2 \log R \sim X^2_{(k)}$ under the assumption that the *k* factors are not associated with time to first recurrence. A factor was added if $P \leq 0.05$; all factors maintained significance once they were included in the model.

Competing risks analyses were completed using the Dynamic 7 PC version of Biomedical Data Package (BMDP, 1993). A log-normal model was used to assess the effects of the covariates on type of first recurrence (local, regional, distant) and time to first recurrence for the whole patient group, as well as for those ≥ 65 years of age. There are three hierarchical hypotheses tested for each covariate: (1) the covariate has no effect on type of first recurrence and time to first recurrence; (2) the covariate has no effect on type of first recurrence; (3) the covariate has no effect on time of first recurrence, given it has no effect on type of first recurrence. A result was significant if $P \leq 0.05$.

### RESULTS

Table 1 indicates that there was a similar pattern of first recurrence by age group ($P = 0.28$): 16% local recurrence for those < 65 years vs. 12% for those ≥ 65 years; with corresponding regional recurrence of 3% and 2% respectively; and respective distant recurrence of 17% and 13%.

Table 2 summarizes the first recurrence rates for patients by subgroup classifications of the investigational factors. Patients with larger tumour sizes and lymph node metastases were more likely to have experienced a first recurrence ($P < 0.001$, in each instance). No other factor exhibited significant univariate differences in recurrence by factor subgroups. Figures 1 and 2 are the disease-free survival plots for tumour size and nodal status.

The same factors were included in the best stepwise models using the Cox (Table 3) and log-normal model-types. For all patients, tumour size and nodal status had negative associations with time to first recurrence (of any type) while adjuvant chemotherapy, age, adjuvant radiation and PgR had positive associations. For patients ≥ 65 years of age, the only factors included in the stepwise multivariate models were nodal status and tumour size which had negative associations with time to first recurrence.

In the competing risks analyses on the data for all ages of patients, there was significant evidence ($P \leq 0.05$) that the factors adjuvant radiation, age, nodal status, tumour size and adjuvant chemotherapy were associated with type of first recurrence or time to first recurrence; the *P*-value for PgR was 0.24. The covariate adjuvant radiation had a significant effect ($P < 0.001$) on type of first recurrence (Figure 3). It was associated with a longer disease-free period before local recurrence, but had no significant effect on regional or distant recurrence. The other factors (age, nodal status, tumour size and adjuvant chemotherapy) did not have significantly different effects on type of recurrence, but were associated with significant effects on time to first recurrence (Figure 4). Older age, pathologically negative nodes, smaller tumours and receiving adjuvant chemotherapy were associated with longer disease-free periods.

In the competing risks analyses for patients ≥ 65 years of age, there were only two factors with significant evidence of association with type or time to first recurrence: nodal status and tumour size. Both of these factors were associated with time to recurrence (Figure 4); negative nodes and smaller tumours were associated with longer disease-free periods.

© Cancer Research Campaign 1999

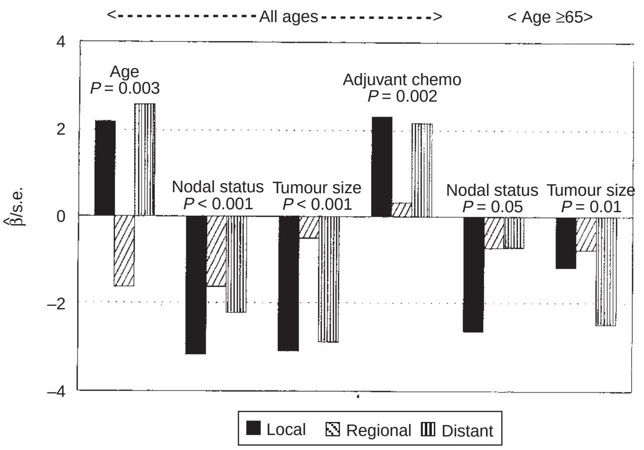

**Figure 4** Covariates with significant effects on time to first recurrence without significant effects on type of first recurrence. The standardized coefficients, $\hat{\beta}$/s.e., permit a comparison of the potential effects across factors. A positive/negative coefficient suggests a positive/negative effect for larger values of the factor on a type of recurrence

### DISCUSSION

Competing risks analyses were developed to assess jointly length of survival for people who have disease-specific recurrence, or die, in a variety of ways (Proschan and Serfling, 1974; Kalbfleisch and Prentice, 1980). Their application to examine type of mortality from breast cancer or other causes may be viewed as straightforward, in the sense that good patient follow-up may produce specific end-points. We have used this technique (Fish et al, 1998) to examine the effects of covariates on the type of death and time to death for this same group of primary invasive breast cancer patients. We found that age was associated with non-breast cancer death; older patients had a greater tendency to die of other causes. Meanwhile, patients with larger tumours were more likely to die from breast cancer. A patient with a high PgR assay value or who had no lymph node involvement was more likely to live longer, without any specific tendency to die either from breast cancer or another cause.

BMDP software provides a simple way of performing competing risks analyses, with the insertion of the statement 'COMP = CRSK' in the regression paragraph of the Survival Analysis program, 2L. Both SAS (SAS, 1988) and S-plus (S-plus, 1991) have programs which fit the accelerated failure time (survival) models; a user may specify the models that should be fit to test the competing risks hypotheses. Allison (1995) describes the process for SAS.

The BMDP software that we used utilizes the model proposed and demonstrated by Lagokos for lung cancer patients (Lagakos, 1978) who could have recurred locally or distantly, or be free of disease ('censored') at the time of investigation. It provides a systematic framework for examining the effects of covariates. There are three hierarchical hypotheses tested for each covariate: (1) the covariate has no effect on type of first recurrence and time to first recurrence; (2) the covariate has no effect on type of first recurrence; (3) the covariate has no effect on time of first recurrence, given it has no effect on type of first recurrence.

The interpretation of our results requires a short discussion at this point. Hierarchical step (2) tests whether there is evidence that the parameters for local, regional and distant recurrence are significantly different. If a test is significant, then one might examine that factor's effect on time to recurrence, by particular type of recurrence. If not, then the overall effect of the covariate on time to first recurrence, of any type, is tested in step (3). The direction of a factor's effect may be different, although not significantly different ($P \leq 0.05$), for one type of recurrence from the others (e.g. see Figure 2 for all ages of patients, where $P = 0.06$ for the test of differences by recurrence type and where the direction of association between age and regional recurrence is opposite to that for age with local or distant recurrence); in this case, the overall effect on time to first recurrence, regardless of type, is examined in step (3). As well, a particular factor may not have a significant effect on a type of recurrence (e.g. see Figure 2 for all ages of patients, the effect of tumour size on regional recurrence is not significant, although it is for local and distant recurrence); again, in this instance, the overall effect on time to first recurrence, regardless of type, will be examined. The complexity of these results, and need for sound clinical interpretation, lead to reporting the full details by type of recurrence, rather than the global test results based on statistical significance.

The log-normal model was chosen for our competing risks analyses because this model choice is well-supported for breast cancer data (Chapman et al, 1996; Gamel et al, 1994, 1995; Rutqvist et al, 1984). The underlying assumption for a log-normal model is that, after a certain point in time, the annual risk of breast cancer recurrence or death will decrease. It would be fairly well accepted from clinical practice that after 3, 5 or 10 years this does happen. Veronesi's data (Veronesi, 1995) demonstrate this decrease in a competing risks context.

For all patients, we found that only adjuvant radiation differentially affected the type of first recurrence. Gelman et al (1990) showed that analytic assumptions about unobtainable recurrence information could lead to difficulties ascertaining whether adjuvant radiation was a beneficial adjunct for the local control of breast cancer, and advocated the use of competing risks to assess the effects of factors. In this context, we confirm with the competing risks analyses the expected clinical importance of adjuvant radiotherapy in prolonging disease-free time for local recurrence; there was no substantial benefit from adjuvant radiotherapy to these patients for regional or distant metastasis.

Adjuvant chemotherapy was associated with increased disease-free time for both local and distant recurrence; the latter is expected clinically, and the former may be attributed to a lack of independence between local and distant recurrence. There was no attributable benefit from adjuvant chemotherapy for regional recurrence.

Overall, older age was associated with increased disease-free time. The non-significant decrease in time to regional recurrence for older patients may reflect a tendency for fewer older women to have received an axillary dissection, and to have needed a delayed axillary dissection outside the primary surgery period (Fish et al, 1998). Veronesi et al (1995) found that a patient's age was an important predictor of local and, to a lesser extent, distant recurrence. We found strong evidence that age was an important predictor for both local and distant recurrence.

We found across all ages of patients that there was significant indication that nodal status and tumour size were predictors of both local and distant recurrence; however, for patients 65 or older, tumour size appeared to be a better predictor of distant, than local

 © Cancer Research Campaign 1999

recurrence while nodal status was a better predictor of local, than distant recurrence. Veronesi et al (1995) observed that tumour size and nodal involvement were important predictors of distant recurrence, but not local recurrence. Any differences between our results and those of Veronesi may be attributed easily to our heterogeneous patient group, lower use of adjuvant radiation, and the consideration of fewer factors. The results of more standard single end-point investigations with a more extensive group of factors and non-competing risks have been reported elsewhere (Chapman et al, 1996, for local recurrence; Pritchard et al, 1993, for distant recurrence).

For the whole group of patients, there were five factors with significant associations with type or time to first recurrence. Only adjuvant radiotherapy was associated with a single type of recurrence: local. Age, nodal status, tumour size and adjuvant chemotherapy all had significant associations across all types of first recurrence, and in particular with time to recurrence for both local and distant metastasis. This suggests a potential lack of independence in these two end-points which many clinicians might not expect.

If distant recurrence was used as a surrogate end-point for mortality, there would only have been partial concordance at this assessment with the competing risks of death results for these same patients (Fish et al, 1998): the factors with significant associations with death were age, tumour size, nodal status and PgR.

The ability to assess covariate effects for a particular type of event is related to the event rate. In this instance, only 21 of the 231 events were due to regional recurrence; this undoubtedly affected the ability to observe significant covariate effects. However, in this instance, the low rate of regional recurrence, especially for the 169 Nx, clinically node negative, patients, is itself an important result; this has been analysed and discussed in depth elsewhere (Fish et al, 1998). It should be noted here that most of the Nx patients did not receive adjuvant radiotherapy, but there was a predominance of elderly in this group. Additionally, tumours tended to be small; mammography has been used routinely at our institution for more than three decades.

In conclusion, we found, as both Gelman et al (1990) and Veronesi et al (1995) did, that it is important to assess the effects of competing types of recurrence for breast cancer patients. Factors may differentially affect the type of recurrence, and the standard assumption of independence may not be appropriate. The availability of commercial packages which can be used to perform competing risks analyses facilitates the use of this approach.

We demonstrated this methodology for traditional factors obtained for a cohort of patients who have long follow-up. The ability to assess simultaneously the effects of new covariates on different types of recurrence will expedite investigations of the relevance for and biologic activity of new prognostic factors.

### ACKNOWLEDGEMENTS

This research was funded by the EB Fish Research Fund. The authors thank David Hallett for technical assistance.

### REFERENCES

Allison PD (1995) *Survival Analysis using the SAS System*: *a Practical Guide* pp 185–209. SAS Institute: Cary, NC

BMDP NC (1993) *Statistical Software, PC Dynamic 7.0*. Statistical Solutions: Saugua, MA

Chapman JW, Hanna W, Kahn HJ, Lickley HLA, Wall J, Fish EB and McCready DR (1996) Alternative multivariate modelling for time to local recurrence for breast cancer patients receiving a lumpectomy alone. *Surg Oncol* **5**: 265–271.

Cox DR and Oakes D (1984) *Analysis of Survival Data*, pp 142–155. Chapman & Hall: London

Fish EB, Chapman JW and Link MA (1998) Competing causes of death for primary breast cancer. *Ann Surg Oncol* **5**(4): 368–375.

Gamel JW, Vogel RL, Valaguisa P and Bonadonna G (1994) Parametric survival analysis of adjuvant therapy for stage II breast cancer. *Cancer* **74**(9): 2483–2490

Gamel JW, Meyer JS and Province MA (1995) Proliferative rate by S-phase measurement may affect cure of breast carcinoma. *Cancer* **76**(6): 1009–1018

Gelman R, Gelber R, Henderson IC, Coleman CN and Harris JR (1990) Improved methodology for analyzing local and distant recurrence. *J Clin Oncol* **8**(3): 548–555

Kalbfleisch JD and Prentice RL (1980) *The Statistical Analysis of Failure Time Data*, pp 21–38, 179–188. John Wiley: New York

Lagakos SW (1978) A covariate model for partially censored data subject to competing causes of failure. *Appl Statist* **27**(3): 235–241

Prentice RL and Marek P (1979) A qualitative discrepancy between censored data rank tests. *Biometrics* **35**: 861–867

Pritchard KI, Trudeau ME, Chapman JW, Hanna W, Kahn HJ, Murray D, Sawka CA, Mobbs BG, Andrulis I, McCready DR and Lickley HLA (1993) Prognostic variables in node-negative breast cancer: an all subset analysis. *Proc ASCO* **12**: 68 (Abstract)

Proschan F and Serfling RJ (eds) (1974) *Reliability and Biometry, Statistical Analysis of Lifelength*. Society for Industrial and Applied Mathematics: Philadelphia, PA

Rutqvist LE, Wallgren A and Nilsson BO (1984) Is breast cancer a curable disease? *Cancer* **53**: 1793–1800

SAS (1993) *SAS/STAT User's Guide*, Version 6, 4th ed., Vol. 2, pp 997–1025. SAS Institute: Cary, NC

S-plus (1991) *S-plus 4 Guide to Statistics*, pp 697–710. Data Analysis Products Division, Math Soft: Seattle, WA

Veronesi U, Marubini E, Del Vecchio M, Manzari A, Andreola S, Greco M, Luini A, Merson M, Saccozzi R, Rilke F and Salvadori B (1995) Local recurrences and distant metastases after conservative breast cancer treatments: partly independent events. *JNCI* **87**(1): 19–27

*© Cancer Research Campaign 1999*
